# Age-Dependent Assortativeness in Herpes Simplex Virus Type 1 Oral Transmission in the United States: A Mathematical Modeling Analysis

**DOI:** 10.1093/infdis/jiaf157

**Published:** 2025-03-26

**Authors:** Hassan Hachem, Houssein H Ayoub, Laith J Abu-Raddad

**Affiliations:** Infectious Disease Epidemiology Group, Weill Cornell Medicine–Qatar, Cornell University, Qatar Foundation–Education City, Doha, Qatar; Department of Healthcare Policy and Research, Weill Cornell Medicine, Cornell University, New York, New York, USA; Mathematics Program, Department of Mathematics and Statistics, College of Arts and Sciences, Qatar University, Doha, Qatar; Infectious Disease Epidemiology Group, Weill Cornell Medicine–Qatar, Cornell University, Qatar Foundation–Education City, Doha, Qatar; Department of Healthcare Policy and Research, Weill Cornell Medicine, Cornell University, New York, New York, USA; Department of Public Health, College of Health Sciences, QU Health, Qatar University, Doha, Qatar; College of Health and Life Sciences, Hamad bin Khalifa University, Doha, Qatar

**Keywords:** oral herpes, genital herpes, assortativeness, Bayesian framework, mathematical model

## Abstract

**Background:**

Herpes simplex virus type 1 (HSV-1) is a highly infectious, globally prevalent lifelong infection. Despite advancements in understanding its epidemiology, the assortativeness in the age-dependent transmission patterns remains unclear. This study aimed to estimate the degree of assortativeness in age group mixing for oral-to-oral HSV-1 transmission within the United States (US) population.

**Methods:**

An age-structured mathematical model was employed to describe HSV-1 transmission dynamics in the US population, incorporating its different modes of transmission. The model was fitted to nationally representative HSV-1 data from the National Health and Nutrition Examination Survey (NHANES) spanning 1976–2016 using a Bayesian inference framework. The degree of assortativeness in age group mixing was calibrated on a scale from 0 (no age group bias in close-proximity interactions) to 1 (exclusive mixing within the same age group).

**Results:**

The model demonstrated robust fits to US demographics, age-specific HSV-1 prevalence, and temporal trends in both HSV-1 prevalence and ever-symptomatic HSV-1 genital herpes prevalence. The degree of assortativeness was estimated as 0.87 (95% credible interval [CrI], .64–.99) for children, indicating strong age-based assortativity, and as 0.04 (95% CrI, .004–.10) for adults, indicating weak age-based assortativity.

**Conclusions:**

Most HSV-1 infections among children are acquired from peers within their own age group, whereas adults acquire HSV-1 infections from a broad range of age groups.

## Teaser

Herpes simplex virus type 1 transmission exhibits distinct age-related patterns, characterized by strong age assortativity among children and weak age assortativity among adults. This distinction highlights the critical role of age-specific social networks in shaping transmission dynamics.

Herpes simplex virus type 1 (HSV-1) is a highly infectious and globally prevalent lifelong infection, typically acquired during childhood [1–3]. The World Health Organization estimated that in 2020, 122.2 million new HSV-1 infections occurred worldwide among individuals aged 0–49 years, contributing to 3779.1 million prevalent infections and a global prevalence of 58.6% in this age group [1]. HSV-1 infection is characterized by frequent subclinical infectious shedding and symptomatic reactivation in a subset of infected individuals [4–6].

The virus is primarily transmitted through contact with cold sores or oral secretions during asymptomatic shedding [5, 7, 8]. This can occur through practices that are particularly common among children, such as sharing utensils, cups, bottles, or food; kissing and other close face-to-face interactions; chewing on shared objects such as toys; and sharing personal items like toothbrushes or lip balm [5, 7, 8].

While symptomatic infection often manifests as oral or facial lesions [9, 10], HSV-1 infection can also cause a wide range of clinical presentations, including herpetic whitlow, gingivostomatitis, meningitis, encephalitis, and corneal blindness [11–13]. The virus can also be transmitted through oral sex or sexual intercourse, resulting in genital herpes infection; however, prior oral HSV-1 infection substantially reduces the risk of acquiring genital HSV-1 [8, 14, 15]. In many high-income countries, improved hygiene and living conditions have reduced the prevalence of oral infections during childhood [3, 8, 16–19], contributing to an increasing trend in the sexual acquisition of HSV-1 [8, 20–23].

Despite advancements in understanding the global epidemiology of HSV-1 infection [8, 20–27], important knowledge gaps persist. One key gap is the limited understanding of the transmission patterns of infection across different age groups. For instance, to what extent do new infections in a specific age group, such as children aged 10–14 years, result from transmission within the same age group versus from other age groups in the population?

This study aims to address this question by estimating the degree of assortativeness in age group mixing for oral-to-oral HSV-1 transmission. A mathematical model of HSV-1 transmission dynamics was employed [8] and calibrated to the United States (US) population—the only country with repeated, nationally representative population-based surveys spanning several decades [3, 16, 28–32], enabling such an analysis. The model was used to describe HSV-1 transmission dynamics across its various modes of transmission and to estimate the degree of assortativeness in age group mixing for both children and adults, accounting for the fact that these groups may have distinct structures of social networks through which the infection is acquired.

## METHODS

### Mathematical Model

The study utilized a deterministic population-level dynamical model previously developed to describe oral and sexual HSV-1 transmission within a given population [8]. The details of the model's structure, equations, and parameterization have been published elsewhere [8] but are briefly summarized here.

The model was constructed based on the current understanding of the natural history and epidemiology of HSV-1 infection [4–6, 8, 33]. It consisted of a set of coupled nonlinear differential equations that stratified the population into compartments based on age, HSV-1 status, stage of infection, and level of oral or genital exposure risk to the infection [8]. Model implementation and analysis were conducted using MATLAB R2019a.

The model accounted for differences in the natural history of HSV-1 infection depending on the route of exposure (oral vs genital) [4–6, 8, 33] and included 2 stages of viral shedding—primary infection and reactivation—as well as periods of latency without viral shedding between reactivation episodes [8]. Susceptible individuals who acquired HSV-1 for the first time progressed through a primary infection phase, followed by a latent infection phase with episodic reactivations throughout their lifetime [4–6, 8, 33].

The population was stratified into 20 age groups, each representing a 5-year age band (0–4, 5–9, …, 95–99 years) [8]. The model accounted for heterogeneity in sexual behaviors across these age groups [8].

### Force of Infection

The force of infection in the model captures 4 distinct age-dependent transmission modes, reflecting the evolving dynamics of HSV-1 epidemiology [8]. Infection acquisition is categorized into 2 primary pathways: oral and genital acquisition. Oral HSV-1 acquisition occurs primarily through oral-to-oral transmission, the dominant mode of infection. A small proportion of oral infections also result from genital-to-oral transmission via oral sex, though this contributes minimally to overall oral HSV-1 acquisition.

Genital HSV-1 acquisition occurs through oral-to-genital and genital-to-genital transmission. Oral-to-genital transmission, through oral sex, accounts for most new genital HSV-1 infections. Meanwhile, genital-to-genital transmission via sexual intercourse serves as a secondary mode of HSV-1 genital acquisition.

The model assumes that children aged <15 years acquire HSV-1 exclusively through oral-to-oral transmission. However, among adolescents and adults (≥15 years), all 4 transmission modes contribute to HSV-1 epidemiology. By incorporating age-dependent variations in transmission risk and behavioral heterogeneity in sexual exposure, the model provides a nuanced representation of HSV-1 transmission dynamics across different life stages.

### Age Group Mixing

The mixing between populations in different age groups—representing the sociophysical interactions that facilitate oral-to-oral transmission of the infection—was characterized using a mixing matrix [34]. This matrix defines the probability of an individual in a specific age group interacting with an individual in another age group [8, 34–36].

The matrix is structured to include 2 components: the first represents strictly assortative mixing, where interactions occur only within the same age group, while the second represents proportionate mixing, where individuals mix with others in the population without preferential bias for a specific age group [8, 34–36]. Proportionate mixing assumes that contact rates between groups are proportional to their relative sizes, resulting in random, homogeneous interactions across the entire population [8, 34–36]. This formulation provides a flexible representation of real-world mixing patterns, balancing age-assortative interactions with broader population-wide exposure.

The matrix is expressed as follows [8, 34–36]:


$$H_{a,b}^{Oral}=\varepsilon_{a}^{Oral}\delta_{a,b}+(1-\varepsilon_{a}^{Oral})\frac{\sum_{i=1}^{n_{Pop}}\left(\rho_{X_{b}^{i}}^{Oral}X_{b}^{i}\right)}{\sum_{u=1}^{20}\sum_{i=1}^{n_{Pop}}\left(\rho_{X_{u}^{i}}^{Oral}X_{u}^{i}\right)}$$

Here, *a* indexes the 20 age groups in the model, and *i* indexes the different population variables ($X_{a}^{i}$) in the model. $\delta_{a,b}$ represents the identity matrix, and $\rho_{X_{a}^{i}}^{Oral}$ ​ is the sociophysical contact rate for the $X_{a}^{i}$ population. The parameter $\varepsilon_{a}^{Oral}$ ​ represents the degree of assortativeness in age group mixing and is defined as:


$$\varepsilon_{a}^{Oral}=\left\{ \begin{array}{c} \begin{array}{cc} \varepsilon_{childhood} & \mathrm{if}\;a<15\mathrm{years}\;\mathrm{age} \end{array}\\ \begin{array}{cc} \varepsilon_{adulthood} & \mathrm{if}\;a\geq 15\mathrm{years}\;\mathrm{age} \end{array} \end{array}\right.$$

Accordingly, there are 2 assortativeness parameters estimated in this study: one for children and one for adults.

This form of the mixing matrix accommodates a spectrum of mixing behaviors, ranging from fully assortative mixing to fully proportionate mixing [8, 34–36]. At one extreme, where $\varepsilon_{a}^{Oral}=1$, mixing is fully assortative, indicating that individuals interact exclusively within their own age group [8, 34–36]. Conversely, at the other extreme, $\varepsilon_{a}^{Oral}=0$, mixing is fully proportionate, signifying no preferential bias in age group interactions [8, 34–36].

Notably, while this formulation of the mixing matrix is flexible and accommodates diverse mixing patterns, it is not exhaustive and does not account for all theoretically possible interactions. Further details on the mixing matrix structure, population variables, and definitions can be found in Ayoub et al [8].

### Data Sources and Model Parameters

The model parameter values were derived from primary studies on HSV-1 natural history, epidemiology, and reported sexual behavior patterns, as detailed previously, including the parameter values, their justifications, and sources [8]. Demographic data, including population size by age, as well as historical and future projections, were obtained from the Population Division database of the United Nations Department of Economic and Social Affairs [37].

A range of epidemiological data was derived from 11 publicly available biennial rounds of the National Health and Nutrition Examination Survey (NHANES), conducted between 1976 and 2016, a nationally representative and population-based survey [3, 16, 28–32]. These data were used to inform model parameters and calibrate the model. Each survey round employed standardized methodologies for both analytical and laboratory procedures [3, 16, 28–32]. Demographic, sexual behavior, and HSV-1 laboratory testing data from each round were extracted, merged, and analyzed in accordance with NHANES standardized guidelines [38].

The laboratory methods distinguished HSV-1 from herpes simplex virus type 2 (HSV-2) antibodies using type-specific serological testing, which detected antibodies against glycoprotein G-1 (gG-1) for HSV-1 and glycoprotein G-2 (gG-2) for HSV-2, ensuring accurate differentiation between the 2 infections. Sampling weights were applied to all NHANES-derived measures to ensure representativeness of the US population.

For each NHANES round, the age-specific distribution of HSV-1 prevalence was derived, as well as the age-specific distribution of self-reported ever-symptomatic and clinically diagnosed genital herpes prevalence in individuals who were concurrently HSV-1 antibody positive and HSV-2 antibody negative [8]. This distinction ensures that genital herpes cases are attributed to HSV-1, as the presence of HSV-1 antibodies and the absence of HSV-2 antibodies indicate that the infection was acquired through HSV-1 rather than HSV-2.

Additionally, the distribution of the reported number of sexual partnerships in the past 12 months was derived, along with the age-specific distribution of sexual partnerships during the same period.

### Model Calibration

The model was first fitted to the population size projections for the US, as provided by the Population Division of the United Nations Department of Economic and Social Affairs [37]. Fitting was performed using a nonlinear least-squares method, implemented with the Nelder-Mead simplex algorithm [39].

Subsequently, the model was fitted to NHANES time-series and age-specific data for HSV-1 prevalence and self-reported ever-symptomatic HSV-1 genital herpes prevalence using a Bayesian inference framework. The prevalence data, reported as proportions bounded between 0 and 1, were modeled using a beta distribution likelihood function.

Let $P_{t}(a)$ denote the model-predicted prevalence for a specific age group *a* at time *t*. The observed prevalence for the corresponding age group and time, $Y_{t}(a)$ ​, was assumed to follow a beta distribution:


$$Y_{t}(a)∼\mathrm{Beta}(\alpha_{t}(a),\beta_{t}(a))$$

where the beta distribution parameters $\alpha_{t}(a)$ and $\beta_{t}(a)$ were determined using the mean and variance of the distribution. The mean was set equal to the model-predicted prevalence, while the variance was derived from the 95% confidence interval of the NHANES-measured prevalence for the corresponding age group and time period.

Seven model parameters were estimated within this Bayesian framework, including $\varepsilon_{childhood}$ and $\varepsilon_{adulthood}$, as well as 5 parameters characterizing the temporal variation in oral exposure risk to HSV-1 infection within the US population, and the overall exposure risk through oral sex. Broad uniform prior distributions were assigned to all parameters to reflect minimal prior information. The priors for $\varepsilon_{childhood}$ and $\varepsilon_{adulthood}$ were assumed to be Uniform(0,1) distributions covering their full possible ranges. The prior distributions for the other parameters were informed by previous fitting of these parameters [8].

The model was solved numerically using MATLAB R2019a to generate the model-predicted prevalence at the observed NHANES time points. Bayesian inference was performed using Markov chain Monte Carlo sampling to generate posterior distributions for the model parameters. The sampling process employed a reversible Markov chain with a stationary distribution corresponding to the target posterior distribution.

The Metropolis-Hastings algorithm [40] was used to estimate the model parameters by iteratively proposing new parameter values from a truncated normal proposal distribution, with the mean set to the current parameter value and truncation ensuring all sampled values were valid. Proposed values were accepted or rejected based on the ratio of the posterior densities. Initial values for the parameters were randomly drawn from their respective prior distributions. Three parallel chains were run, each initialized independently, and the first half of the iterations were discarded as burn-in [41]. The algorithm was executed for a sufficient number of iterations to ensure convergence.

Parameter estimates were calculated as the means of the posterior samples, with 95% credible intervals (95% CrIs) used to quantify uncertainty. Convergence diagnostics, including the Rhat statistic (Gelman-Rubin diagnostic), effective sample size, and autocorrelation, were evaluated to ensure proper convergence. Posterior predictive checks were performed to assess the agreement between model-predicted prevalence and observed prevalence data. Accordingly, this Bayesian calibration framework facilitated robust parameter estimation while accounting for the variability and bounded nature of the observed prevalence data.

Notably, NHANES HSV-1 surveys did not include children aged 0–4 years. Consequently, model calibration to these data cannot account for mixing patterns within this age group, limiting the model's applicability to older children.

## Oversight

This study is based on mathematical modeling using publicly available data; therefore, no patient consent or ethical approval is required.

## RESULTS

**Figure 1.**
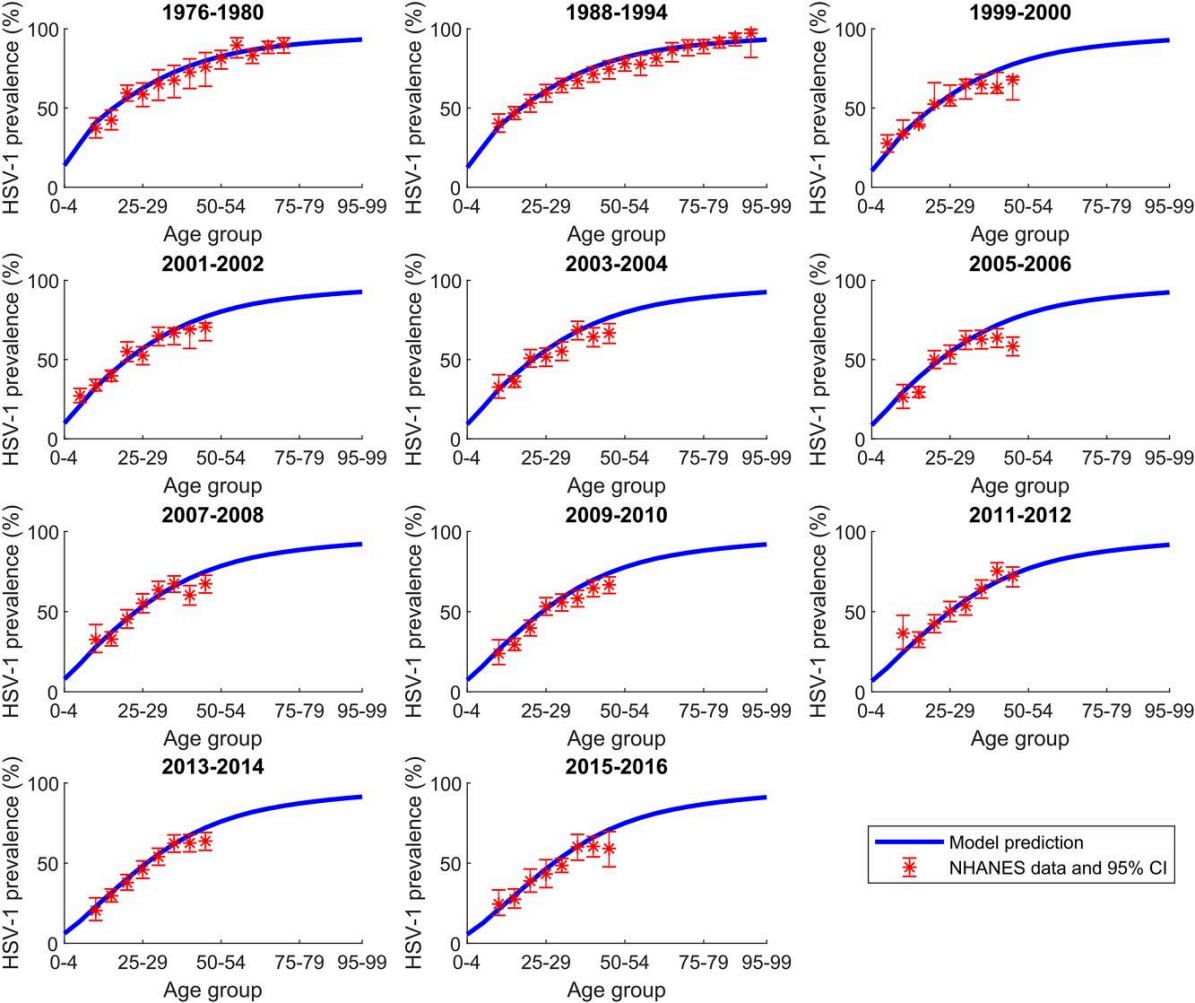
Fitting of age-specific herpes simplex virus type 1 (HSV-1) prevalence for each National Health and Nutrition Examination Survey (NHANES) round. Comparison of the model-fitted HSV-1 prevalence for each 5-year age band in the United States with NHANES data from 1976 to 2016. Abbreviations: CI, confidence interval; HSV-1, herpes simplex virus type 1; NHANES, National Health and Nutrition Examination Survey.

**Figure 2.**
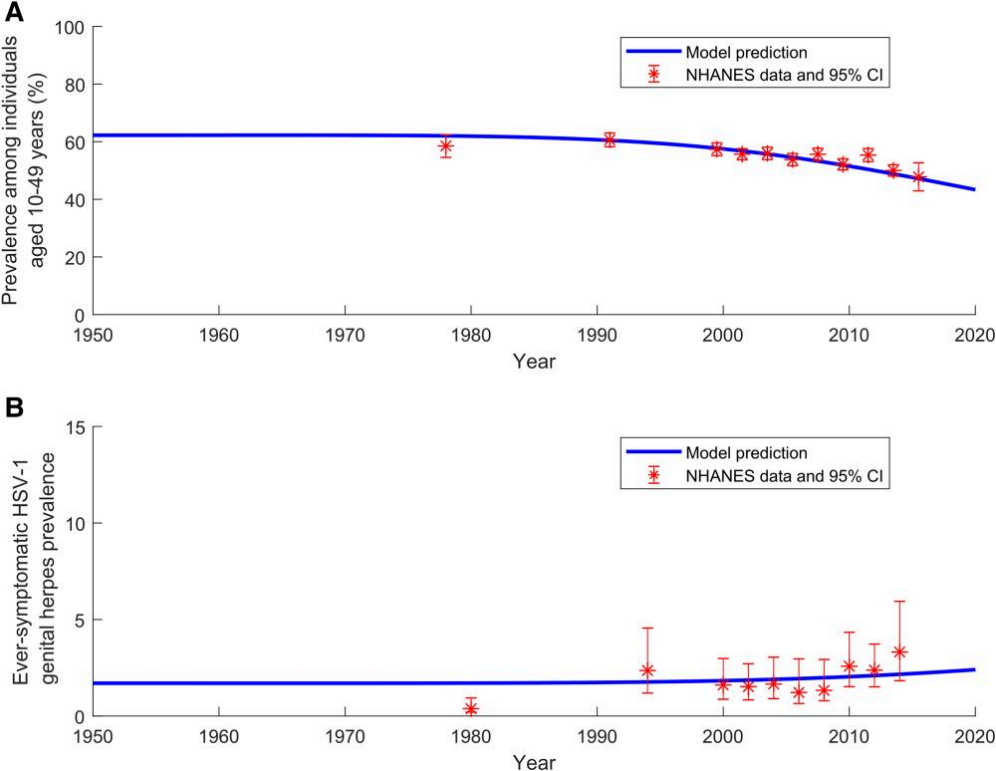
Fitting of herpes simplex virus type 1 (HSV-1) prevalence temporal evolution. *A*, Comparison of the model-fitted temporal trends in HSV-1 prevalence among individuals aged 10–49 years in the United States (US) with National Health and Nutrition Examination Survey (NHANES) data. *B*, Comparison of the model-fitted temporal trends in ever-symptomatic HSV-1 genital herpes prevalence among individuals aged 20–49 years in the US with NHANES data. Abbreviations: CI, confidence interval; HSV-1, herpes simplex virus type 1; NHANES, National Health and Nutrition Examination Survey.

The model demonstrated robust fits to US population size (Supplementary Figure 1), age-specific HSV-1 prevalence data across all NHANES rounds (Figure 1), the temporal trend of HSV-1 prevalence (Figure 2*A*), and the temporal trend of ever-symptomatic HSV-1 genital herpes prevalence (Figure 2*B*).

The modeled transmission dynamics indicate a sustained decline in both the HSV-1 oral force of infection and HSV-1 prevalence over the past few decades (Figure 2*A*). As a result, the age at which HSV-1 prevalence reaches 50% has steadily increased (Figure 1), shifting from the age group 20–24 years in 1990 and 2000 to 25–29 years in 2010 and 35–39 years in 2020.

**Figure 3.**
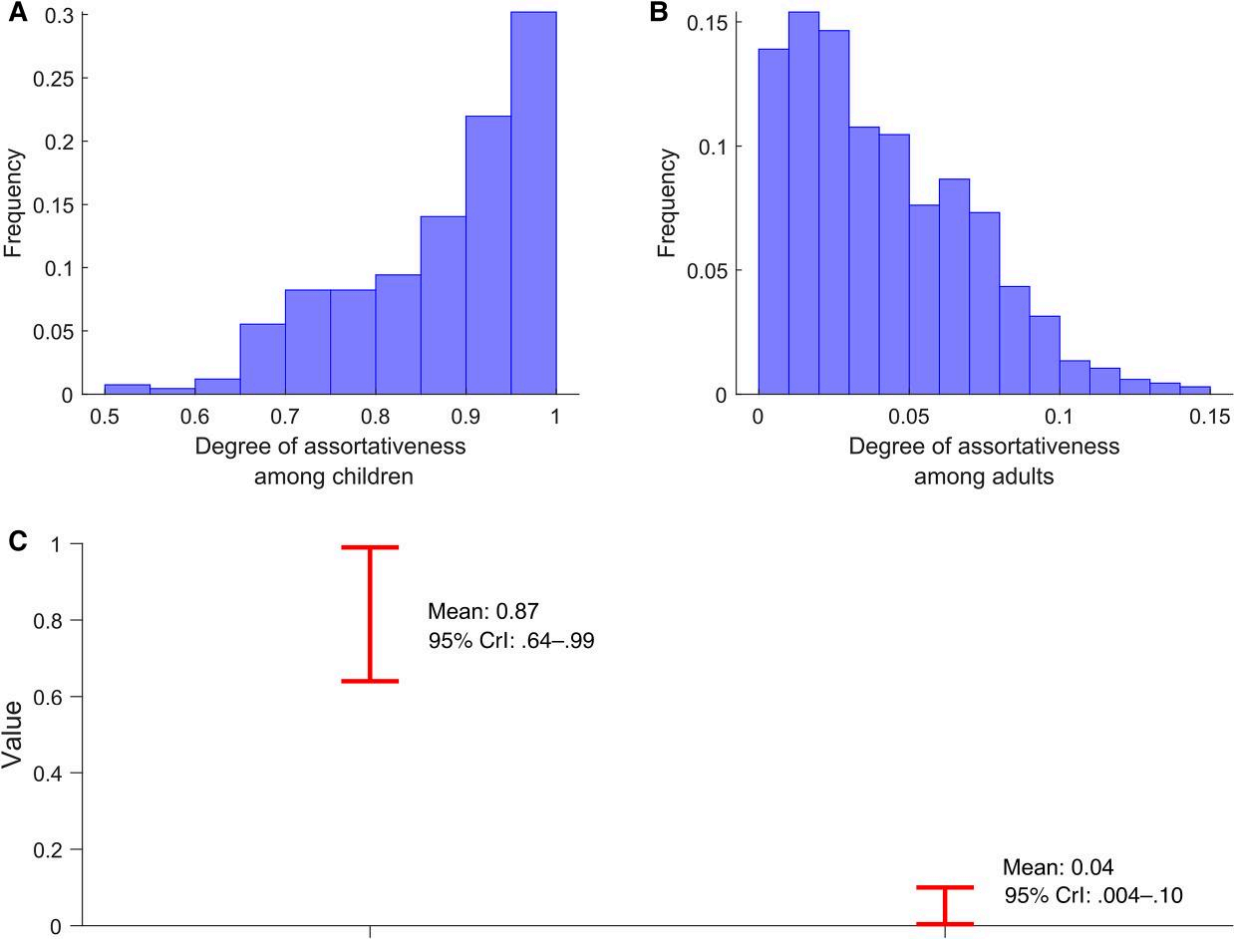
Degree of assortativeness in herpes simplex virus type 1 infection transmission among children and adults. *A*, Model-estimated distribution of the degree of assortativeness among children aged 0–14 years. *B*, Model-estimated distribution of the degree of assortativeness among adults aged 15–99 years. *C*, Model-estimated mean and 95% credible interval (CrI) for the degree of assortativeness among children and adults.

Figure 3 *A* and 3*B* show the model-estimated distributions for the degree of assortativeness among children aged 0–14 years and adults aged 15–99 years, respectively. The model estimated the degree of assortativeness for children ($\varepsilon_{childhood}$​) as 0.87 (95% CrI, .64–.99) and for adults ($\varepsilon_{adulthood}$​) as 0.04 (95% CrI, .004–.10) (Figure 3*C*).

Supplementary Figure 2 illustrates the distributions of the 5 additional model parameters estimated in this study. These parameters include those characterizing the variation in exposure risk to oral HSV-1 infection within the US population over time: *Z*, $\rho_{X(a,i)}^{Oral}$, $\xi_{Turning}$, and $\xi_{Duration}$ (Supplementary Figure 2*A–D*), as well as the overall exposure risk through oral sex: $C^{OralSex}$ ​ (Supplementary Figure 2*E*). The figure also presents the mean estimates and their corresponding 95% CrIs. Definitions and details on these parameters are available in Ayoub et al [8].

## DISCUSSION

This study provides evidence of age-related patterns in HSV-1 transmission, revealing distinct dynamics between children and adults. Among children, specifically those older than 5 years, HSV-1 transmission exhibits strong age assortativity, with most infections acquired from peers within the same age group. This pattern suggests that age-specific interactions, such as close-contact activities and shared environments—including daycare centers, schools, and playgrounds—play a major role in sustaining HSV-1 transmission in this demographic. In contrast, for younger children, existing literature indicates that household contacts, particularly parents, are the primary source of HSV-1 transmission [42].

In contrast to children, HSV-1 transmission among adults is weakly assortative by age group. HSV-1 acquisitions in adults are distributed across transmissions from a wide range of age groups, with no single age group predominating as the primary source of viral transmission. This pattern suggests that adult transmission dynamics are driven by more heterogeneous interaction patterns, influenced by diverse social and behavioral factors. These factors may include varied social, occupational, and lifestyle interactions that bridge age groups and facilitate cross-age group exposure.

The observed lack of age-assortative mixing among adults in HSV-1 oral transmission contrasts with the strong age-assortative patterns seen in the sexual transmission of sexually transmitted infections [43, 44]. In the latter, men within a specific 5-year age group typically preferentially mix with women in the adjacent younger 5-year age group [43, 44].

Since viruses such as HSV-1 propagate through social networks, their transmission patterns offer insights into the underlying structures of these networks. The findings suggest that, unlike adults, children's social networks are high-density, age-dependent systems characterized by typical small-world properties [45]. These small-world properties include high clustering, indicating tightly connected groups, and short path lengths, while still maintaining some long-range connections, which enable efficient transmission of infection while balancing local clustering with global reach. These networks may also exhibit a relatively homogeneous distribution of both interaction time and interaction partners, reflecting the structured and frequent interactions typical of children's social environments.

These findings may have implications for other infections transmitted through close-proximity interactions and propagated within social networks. The results align with evidence on influenza transmission patterns, such as in school settings, where strong assortative mixing is facilitated by the structural organization of schools into classes and grades [46–48]. These findings emphasize the role of age-specific interactions in shaping the transmission dynamics of such infections and underscore the importance of tailored public health interventions.

For children, targeted interventions focusing on specific environments such as schools or daycare centers may be most effective, while for adults, broader community-level strategies may be better suited for controlling the spread of such infections. Notably, HSV-1 vaccination, once available, could serve as the most effective public health intervention against HSV-1 infection [49, 50], as it would provide protection regardless of social context, interactions, or age.

This study has limitations. First, NHANES surveys did not include children aged 0–4 years, and only 2 surveys included children aged 5–9 years. As a result, the estimated assortativeness $\varepsilon_{childhood}$ is primarily informed by data from the age group 10–14 years, which was consistently available across all survey rounds. Consequently, $\varepsilon_{childhood}$ estimate is most representative of the age group 10–14 years and does not reflect assortativeness in mixing among younger children aged 0–4 years.

Second, a population-level model was used to examine the degree of age-assortative mixing; however, this type of model parameterizes interactions between age groups collectively and does not explicitly account for household interactions or prolonged household contacts, such as the extended duration of parent–child relationships or sibling interactions at home, which are particularly important during the first few years of life before children enter daycare or school [42]. While an individual-based model could capture such effects, its implementation would still require detailed household-level data on interactions and HSV-1 biomarkers for proper calibration, which are currently unavailable. Notably, as HSV-1 prevalence continues to decline in the broader population [3, 8, 16], decreasing maternal and paternal HSV-1 prevalence may reduce parent-to-child transmission, potentially reinforcing age-assortative mixing patterns in HSV-1 transmission among children.

Third, the mixing matrix was parameterized using a conventional approach [8, 34–36], separating mixing into 2 distinct components: an assortative component based on age and a nonassortative component where mixing occurs without age preference. While this formulation is flexible and accommodates diverse mixing patterns, it is not exhaustive and does not capture other theoretically possible patterns, such as preferential assortative mixing between different age groups, for example, between mothers and their infants.

The goal of this analysis, however, is not to model all potential forms of mixing exhaustively—an approach that is not feasible given the available data for model calibration—but rather to identify a clear signature of variable assortativeness between children and adults. The markedly distinct assortativeness values observed for these 2 groups provide compelling evidence for differing transmission dynamics between children and adults.

Fourth, this study investigated HSV-1 transmission patterns within the US population, which may limit the generalizability of the findings to other populations. However, the observed patterns appear to stem from basic features of social networks that could be consistent across human populations, suggesting that the findings may be applicable, at least in part, to other contexts.

Fifth, the model did not explicitly stratify the population by sex [8]. However, global analyses of HSV-1 epidemiology indicate no significant differences in exposure risk between females and males [20, 24–27], making this limitation unlikely to affect the results.

Finally, the model assumes that HSV-1 infectiousness does not vary with the presence of clinical symptoms and does not account for the potential impact of antiviral treatment during symptomatic episodes or suppressive therapy [8]. While these are simplifications, their effect on the results is expected to be minimal, as the majority of infectious viral shedding occurs asymptomatically and HSV-1 infection is rarely treated [4–6].

This study has strengths. First, to the best of our knowledge, it is the first to investigate assortativeness in the age-dependent transmission patterns of HSV-1 infection. Second, the study utilized standardized, population-based data from NHANES spanning over 4 decades, providing reliable and representative estimates that reflect the sociodemographic diversity and modes of acquisition within the population [3, 16, 28–32]. The use of such representative, statistically precise input data minimized uncertainty in the results and estimates.

Third, a sophisticated mathematical model was employed to capture the complex dynamics of HSV-1 transmission, grounded in high-quality data on the natural history and transmission parameters of HSV-1 infection [8]. Finally, the model demonstrated a robust fit to empirical data, with predicted trends closely aligning with observed patterns. This alignment supports the robustness of the model and the reliability of its findings.

In conclusion, this study identified distinct age-related patterns in HSV-1 transmission, characterized by strong age assortativity among children >5 years of age and weak assortativity among adults. These findings highlight the role of age-specific social networks in shaping transmission dynamics and may have implications for other infections transmitted through close-proximity interactions. These insights support the importance of targeted public health interventions, with strategies tailored to structured environments for children and broader community-level approaches for adults.

## Supplementary Material

jiaf157_Supplementary_Data

## Supplementary Data

Supplementary materials are available at *The Journal of Infectious Diseases* online (http://jid.oxfordjournals.org/). Supplementary materials consist of data provided by the author that are published to benefit the reader. The posted materials are not copyedited. The contents of all supplementary data are the sole responsibility of the authors. Questions or messages regarding errors should be addressed to the author.

## Notes

***Acknowledgments.*** The authors are grateful for infrastructure support provided by the Biomedical Research Program and the Biostatistics, Epidemiology, and Biomathematics Research Core at Weill Cornell Medicine–Qatar. The authors are further grateful for the administrative support of Ms Adona Canlas. ChatGPT was exclusively utilized to verify grammar and refine the English phrasing in our text. No other functionalities or applications of ChatGPT were employed beyond this specific scope. Following the use of this tool, the authors thoroughly reviewed and edited the content as necessary and take full responsibility for the accuracy and quality of the publication.

***Author contributions.*** Conceptualization: L. J. A. Methodology and model development and parameterization: H. H., H. H. A., and L. J. A. Software and analysis: H. H. and H. H. A. Validation and investigation: H. H., H. H. A., and L. J. A. Writing—original draft: H. H. and L. J. A. Writing—review and editing: All authors. Supervision: L. J. A. and H. H. A.

***Data availability.*** Data generated or analyzed during this study are included in the main text and Supplementary Material. The MATLAB codes used for the model can be obtained by contacting the authors.

***Disclaimer.*** The funder had no role in the design and conduct of the study.
