# Supplementary material for: Age-Dependent Assortativeness in Herpes Simplex Virus Type 1 Oral Transmission in the United States: A Mathematical Modeling Analysis
Source: J Infect Dis. 2025 Mar 26;231(6):e1151–9. doi: 10.1093/infdis/jiaf157 (PMC12247797; doi:10.1093/infdis/jiaf157)
Supplement: jiaf157_Supplementary_Data [file jiaf157_supplementary_data.docx]

**Supplementary Material**

**Table of Contents**

[**Figure S1. United States demographics.** Model-estimated total population size in the United States compared to projection of the Population Division of the United Nations Department of Economic and Social Affairs [1]. 2](#_Toc192543718)

[**Figure S2. Model-estimated distributions derived using the Bayesian inference framework.** Distributions of the five other model parameters estimated in this study, including those characterizing temporal variation in oral exposure risk to HSV-1 infection within the United States population and the overall exposure risk through oral sex. Further details on these parameters are available in Ayoub et al. [2]. 3](#_Toc192543719)

[**References** 4](#_Toc192543720)

# **Figure S1. United States demographics.** Model-estimated total population size in the United States compared to projection of the Population Division of the United Nations Department of Economic and Social Affairs [1].


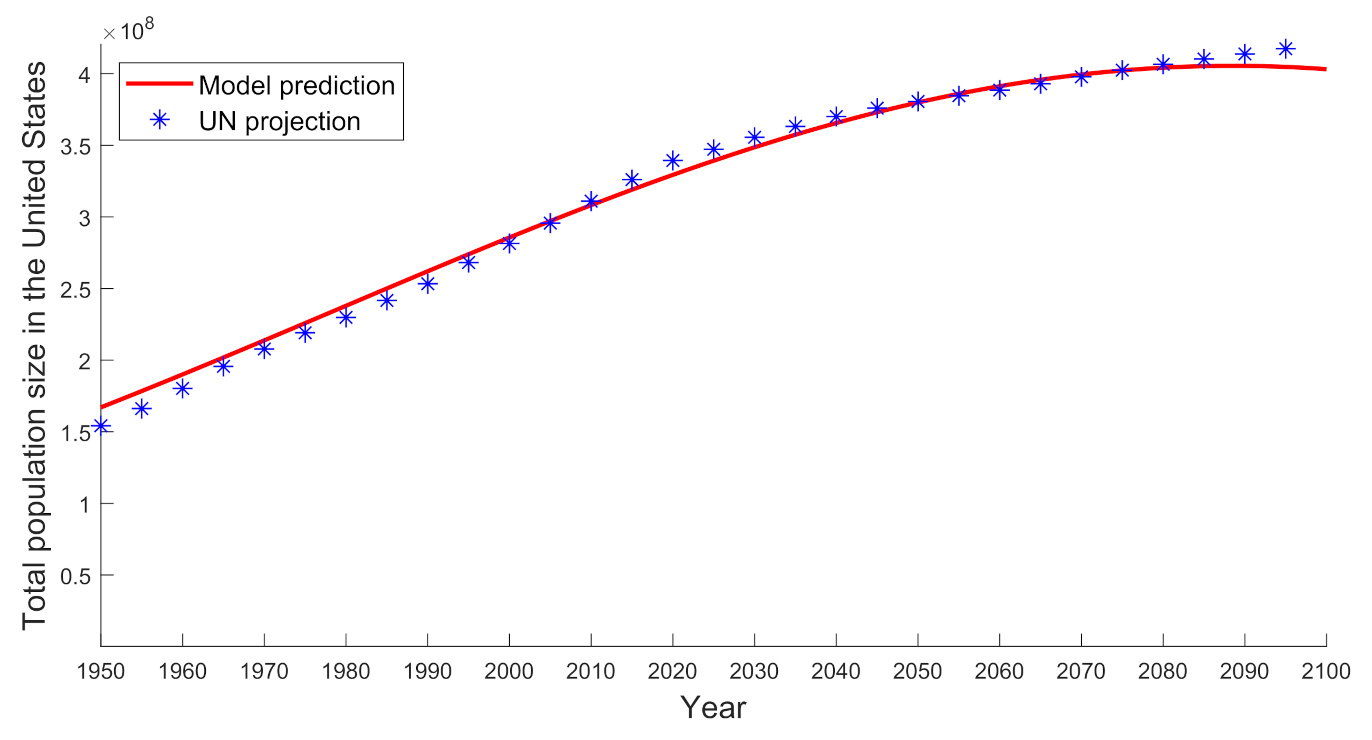


# **Figure S2. Model-estimated distributions derived using the Bayesian inference framework.** Distributions of the five other model parameters estimated in this study, including those characterizing temporal variation in oral exposure risk to HSV-1 infection within the United States population and the overall exposure risk through oral sex. Further details on these parameters are available in Ayoub et al. [2].


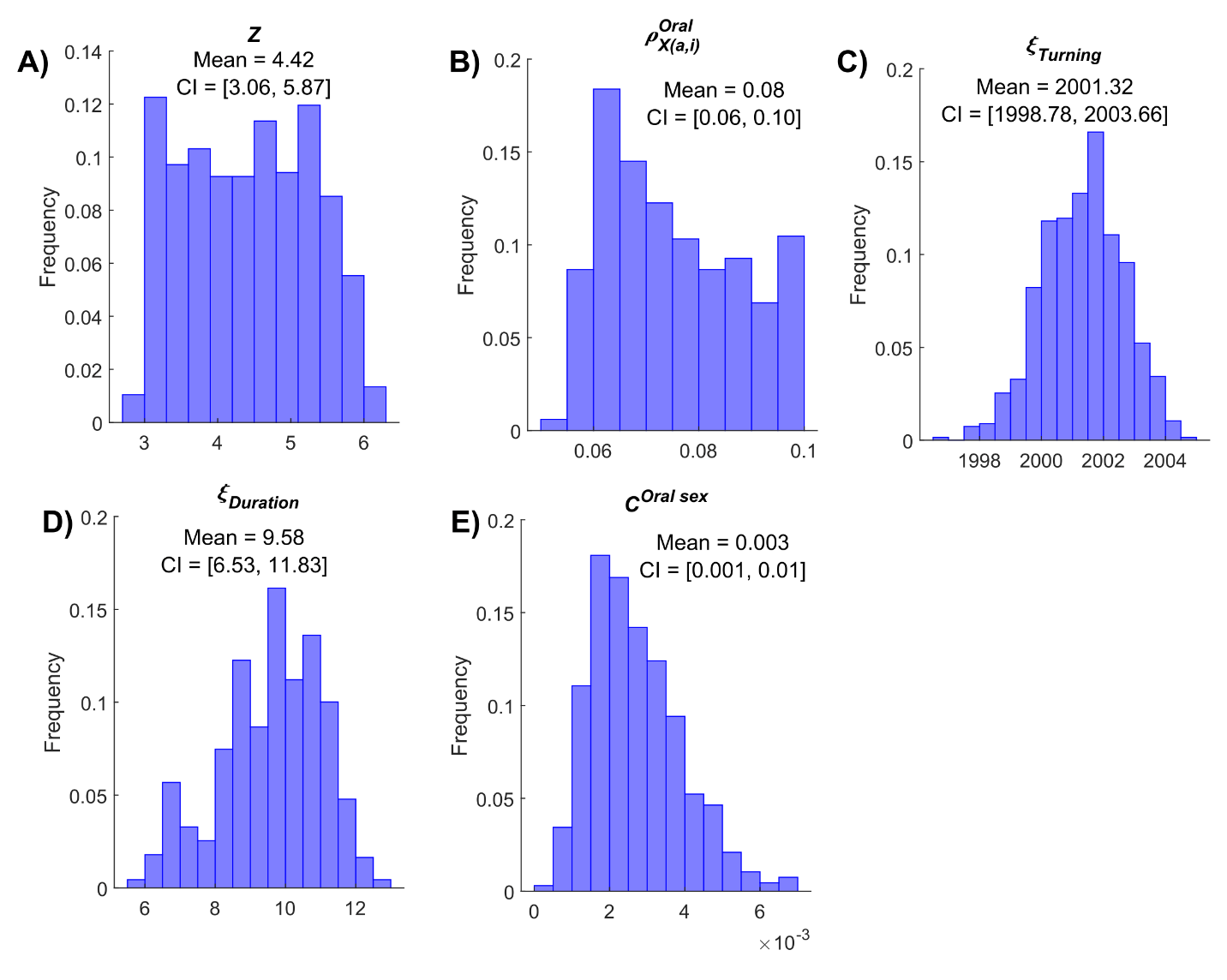


# **References**

1. United Nations Department of Economic and Social Affairs. World Population Prospects, the 2024 Revision, **2024**.

2. Ayoub HH, Chemaitelly H, Abu-Raddad LJ. Characterizing the transitioning epidemiology of herpes simplex virus type 1 in the USA: model-based predictions. BMC Med **2019**; 17:57.
